# Can an educational intervention in the context of inpatient pulmonary rehabilitation improve asthma self-management at work? A study protocol of a randomized controlled trial

**DOI:** 10.1186/s12890-024-02847-8

**Published:** 2024-01-17

**Authors:** Julia Salandi, Markus C. Hayden, Katherina Heinrichs, Matthias Limbach, Konrad Schultz, Gabriele Schwarzl, Wolfgang Neumeister, Adrian Loerbroks

**Affiliations:** 1 Institute of Occupational, Social and Environmental Medicine, Centre for Health and Society, Faculty of Medicine, University of Duesseldorf, Duesseldorf, Germany; 2 Clinic Bad Reichenhall, Centre for Rehabilitation, Pneumology and Orthopaedics, Bad Reichenhall, Germany; 3 Institute of Health and Nursing Science, Charité – Universitätsmedizin Berlin, corporate member of Freie Universität Berlin and Humboldt-Universität zu Berlin, Berlin, Germany; 4 Hufeland-Clinic Bad Ems GmbH, Centre for Pneumology, Bad Ems, Germany

**Keywords:** Asthma, Asthma control, Randomized controlled trial, Rehabilitation, Self-efficacy, Self-management, Quality of life, Work

## Abstract

**Background:**

Asthma self-management (e.g., trigger avoidance or correct medication use) is a cornerstone of therapy. Its successful implementation in everyday working life is determined by psychosocial working conditions, in particular by support from superiors and colleagues and the job decision latitude (i.e., when and how to carry out which tasks). To empower individuals with asthma to modify their working conditions, employees need to use certain communication skills and acquire specific knowledge. Both could be taught as part of patient education during pulmonary rehabilitation. Therefore, the aim of the planned study is the development and multicentre implementation of an education module for individuals with asthma during their rehabilitation and to generate evidence on its effectiveness.

**Methods:**

Participants (*n* ≥ 180) will be recruited, randomized into an intervention and a control group, trained and surveyed in two rehabilitation clinics. The intervention group will receive the supplementary patient education module “Asthma and Work” while the control group will participate in a program on " Eating behaviour” (both 2 × 50 min). The effectiveness of the intervention will be examined based on pre-post measurements (T1 and T2) and a 3-month follow-up (T3). We will consider behavioural intention (T2) and asthma self-management at work (T3) as primary outcomes. Secondary outcomes will include self-management-related knowledge, self-efficacy, number of sick days, number of exacerbations, asthma control (Asthma Control Test), asthma-related quality of life (Marks Asthma Quality of Life Questionnaire), and subjective employment prognosis (Brief Scale Measuring the Subjective Prognosis of Gainful Employment). The pre-post comparisons are to be evaluated using univariate analyses of covariance.

**Discussion:**

Improving asthma self-management at work could increase the work ability and social participation of employees with asthma. This could reduce costs, e.g. in terms of asthma-related sick leave.

**Trial registration:**

German Clinical Trials Register (ID: DRKS00031843)

**Supplementary Information:**

The online version contains supplementary material available at 10.1186/s12890-024-02847-8.

## Background

Self-management of asthma (e.g., trigger avoidance, respiratory physiotherapy techniques for shortness of breath or irritable cough, correct application of medication based on a written therapy and emergency plan, and communication about the disease) is an important component of therapy [1–3]. Qualitative and quantitative findings suggest that beneficial psychosocial working conditions are associated with better asthma self-management at work [4, 5], but also with improved asthma control, asthma-related quality of life, and the subjective employment prognosis (including the personal assessment of the ability to continue working until retirement age, the risk to earning capacity and the intention to apply for a pension) [6, 7]. Among the psychosocial working conditions relevant to asthma self-management, social support (from colleagues and superiors) [4, 5] and decision latitude at work (i.e., the extent to which work tasks and working hours can be determined at one’s own discretion) represent key factors [4]. Communication skills and comprehensive education about relevant patient and employee rights may enable patients to improve these psychosocial working conditions– and thereby, among other things, their asthma self-management [4].

Patient education in the context of pulmonary rehabilitation could be an important approach to empower patients with asthma to modify their workplace in a self-determined manner with consideration of their condition [8]. This endeavour could be understood in terms of the concept of “job crafting”, which assumes that employees can proactively redesign their work situation [9, 10]. It thus implies that an individualized, bottom-up approach to job redesign is possible, as opposed to top-down approaches and organization-initiated “one-size-fits-all” solutions [9, 11, 12]. Even employees in presumably rigid work environments and low-autonomy workplaces appear to have the ability to make some changes to their work demands and resources [9].

In Germany, individuals with asthma mostly receive inpatient pulmonary rehabilitation (duration: about three weeks). Currently, rehabilitation clinics in Germany often schedule only 15 min for patient education on the topic “asthma at work” - along with the complex topics “coming out as an asthmatic” and “asthma in the family” [13]. The aim of the planned multicentre intervention study is to develop and implement a novel patient education module addressing “Asthma and Work” in more depth in pulmonary rehabilitation. The effectiveness of the module will be examined in a randomized controlled trial (RCT) with a follow-up period of 3 months.

In the new patient education module, a change in working conditions is to be achieved by initially increasing knowledge and raising awareness of the topic [3]. However, research has shown that the increased knowledge alone does not translate into behavioural change among patients, but that there are factors that mediate the translation of knowledge into behaviour [14]. Therefore, based on the Theory of Planned Behaviour (TPB), [15–17] we assume that the intention to actually perform a certain behaviour (hereafter referred to as “behavioural intention”) is the most proximal precursor of the behaviour itself (here: asthma self-management at work). An important determinant of the behavioural intention is, in turn, an individual’s confidence in having sufficient skills and in one’s abilities to perform a given behaviour [15, 18, 19]. In the TPB, this concept is referred to as perceived behavioural control and is based on Bandura’s self-efficacy theory [20]. Therefore, in addition to knowledge transfer, we aim to increase job-related self-efficacy, defined here as the confidence that patients feel able to implement their own desired self-management strategies at work, through social skills training (e.g., role-play).

We hypothesise that the behavioural intention related to the implementation of appropriate asthma self-management will improve at the end of the rehabilitation program (T2). As mentioned above, the behavioural intention is considered a key determinant of successful asthma self-management at work [15, 20]. Then, after 3 months and the possible return to work (T3), actual asthma self-management at work should have improved due to prior proactive initiation of changes of relevant individual working conditions. Thus, behavioural intention will be used as primary outcome at T2 while asthma self-management will serve as primary outcome at T3. Self-management-related knowledge and self-efficacy along with other factors (see below) will represent the secondary outcomes. The potential improvement in asthma self-management at work and related improvement in asthma control, asthma-related quality of life, and the subjective employment prognosis (see above) [6, 7] could then reduce the number of exacerbations and sick days (and also related costs). Therefore, we will also examine asthma control, asthma-related quality of life, the subjective employment prognosis, and the number of exacerbations and sick days as secondary outcomes.

## Methods/design

### Study design and data collection

A randomized-controlled intervention study will be carried out comparing the intervention group (patient education “Asthma and Work”) with a control group (patient education “Eating behaviour”, parallel group). We estimate that in total about 180 individuals with asthma will participate in two rehabilitation clinics in Germany (Hufeland-Clinic Bad Ems: *n* ≥ 60, Clinic Bad Reichenhall: *n* ≥ 120) over a period of 15 months (recruitment, implementation, 3-month follow-up). Initially, screening for eligibility for participation takes place approximately four weeks before the start of inpatient pulmonary rehabilitation (T0). Furthermore, measurements at the beginning (T1) and at the end of rehabilitation (T2, about three weeks after T1) as well as a follow-up survey (T3, about three months after T2) are planned (see also Table 1). The data are collected primarily by using a patient questionnaire (self-reports). In addition, medical data are collected at T1 and T2 by the rehabilitation staff (physician questionnaire). The study is coordinated at the University of Duesseldorf, Germany, by the study coordinator (JS) and the study director (AL) (e.g., development of the intervention and control module, study materials, data management and statistical analysis, access to the final dataset). On-site study procedures are carried out by study assistants (e.g., recruitment, management of assignment lists of ID and contact data, documentation of drop-outs or deviations from intervention protocol, a reminder to participate at T3 by phone), and patient education is facilitated by (pre-trained) psychologists. To ensure that our study protocol reports all relevant content, we applied the SPIRITreporting guidelines [21]. The study was registered in the German Clinical Trials Registry (ID: DRKS00031843) on 17.10.2023.

**Table 1:** Time schedule

2023					2024												2025			
Aug	Spt	Oct	Nov	Dec	Jan	Feb	Mar	Apr	May	Jun	Jul	Aug	Spt	Oct	Nov	Dec	Jan	Feb	Mar	Apr
1	2	3	4	5	6	7	8	9	10	11	12	13	14	15	16	17	18	19	20	21
Preparation intervention and control module, development of education materials																				
					Screening (T0) + recruitment															
						Pre-test^1^														
						Implementation phase (T1 + T2)														
							3-month follow-up (T3)													
							Data management and analyses													
													Paper							

^1^Month 1 of the intervention period will serve as a pre-test: There will be two rounds of feedback from the teaching psychologists to the cross-site team (approximately after 2 and 4 weeks). If necessary, adjustments to the education modules will take place afterwards

For ethical reasons, participants are informed about the general scope and contents of both patient education modules. However, a cover story (in the study information we describe a trial run of two new patient education modules to further improve rehabilitation) is intended to prevent participants from knowing which patient education represents the intervention and which the control module. This blinding can be removed at the request of the participants at the earliest after the study has been completed. The therapists who will conduct the patient educations cannot be blinded, as they will already be involved in the design of the educational modules and thus will be aware of the study content and objectives. However, it is planned that the person (JS) who will conduct the statistical analysis in the Institute for Occupational, Social and Environmental Medicine (IASUM) at the University of Duesseldorf will be blinded, i.e. the variable revealing group membership will not be made available to JS until the analysis is completed.

### Patient selection

Four weeks before rehabilitation, all patients with the primary diagnosis of asthma will be contacted by mail and asked to complete the screening questionnaire. Patients will be included in the study if they meet the following criteria (see also Table 2), which are assessed using the screening questionnaire (and partially verified by a physician questionnaire at the start of rehabilitation). All patients receive the usual rehabilitation programme, regardless of whether they take part in the study or not.

1. Referral diagnosis “bronchial asthma”: diagnosis before the start of the pandemic (before March 2020)^1^ + confirmation of the diagnosis (ICD-10 code J45) in the rehabilitation clinic by a pulmonologist based on clinical and lung function diagnostics with spirometry/body plethysmography at T1 (forced expiratory volume in 1 s [FEV1], FEV1/vital capacity [VC], total specific airway resistance [sR_tot_], VC, residual volume [RV]), supplemented by allergy screening (total immunoglobulin E [Ig E], specific Ig E-screening), assessment of exercise capacity (6-minute walk test) and asthmatic inflammation (fractional exhaled nitric oxide [FeNO], eosinophils/µl)
2. Employment in an employee or civil servant relationship subject to social insurance with at least 15 h of work per week.
3. Worked with the asthma diagnosis for at least six months.

Individuals with referral diagnosis of Chronic Obstructive Pulmonary Disease (COPD) or long/post COVID (longer-term, adverse health effects following SARS-CoV-2 infection) are excluded. Mixed leading asthma and COPD cases are accepted as long as asthma is the referral diagnosis. Similarly, individuals with additional self-reported persistent long/post COVID symptoms (e.g., fatigue) remain in the study, but– like individuals with mixed asthma-COPD– are excluded in a sensitivity analysis (see below). If all inclusion criteria are met, study participation is usually only discontinued at the participant’s request.

### Randomization

Randomization is to be performed using a random number table created with computer software (i.e., computer-assisted sequence generation). The randomization lists are created in blocks (block size varies randomly) at the IASUM (outside the clinics, central randomization) for both clinics separately (stratified randomization by study centre). Opaque, sealed envelopes numbered consecutively are sent by the IASUM to the clinics, which contain information on group membership according to the randomization list and which are to be opened in ascending order as soon as a participant is to be randomized. A study assistant at the beginning of rehabilitation will randomly assign all patients who meet the inclusion criteria and have provided informed consent to the intervention or control group. The study assistants on site (concealed allocation) do not know the size of the different blocks. This procedure is in line with current quality standards [22] and has already proven to be feasible and practicable in a large intervention study at the participating Clinic Bad Reichenhall [23].

### Sample size

We assume that over a period of 11 months, we will be able to recruit approximately 300 individuals who meet the inclusion criteria (Bad Ems: *n* ≥ 100, Bad Reichenhall: *n* ≥ 200). With a drop-out rate of 40% by T3, we can assume a sample of 180 participants (90 per group) attending at all measurement time points (Bad Ems: *n* ≥ 60, Bad Reichenhall: *n* ≥ 120). With an intermediate effect size of Cohen’s d = 0.5 [24], an alpha level of 0.05, and a statistical power of 0.8, this sample size should be sufficient to detect significant differences and the sample size is comparable to or exceeds that of similar RCTs [25–28].

### Intervention

Both groups will receive a 3-week rehabilitation program that meets the structural requirements of German healthcare insurance providers (standards for personnel, space, and medical-technical equipment) [29, 30]. The rehabilitation program includes, among other things, different non-drug therapy components such as physical training, respiratory physiotherapy, comprehensive patient education, and psychosocial counselling [31]. In addition, patients will receive a routine check-up and if necessary, the current asthma medication will be adapted according to the current guidelines (any changes to medication will be documented).

The participants of the intervention group will additionally receive the module “Asthma and Work” in a workshop with preferably about five participants each. This education module spans across 2 × 50 minutes. It offers in the first part (module A) a theoretical introduction to the topic/knowledge transfer in the form of lectures (e.g., “What is asthma self-management in general and in the workplace?” and “What does ‘job crafting’ mean?“) as well as individual and plenary exercises (peer support). In the second part (module B), the focus is on practical exercises, especially in the form of communication training and role plays (e.g., “How do I communicate specific change requests regarding my working conditions?”).

The control group, by contrast, will receive an education module addressing “Eating behaviour”. We chose this topic because eating behaviour is not part of asthma self-management behaviour. In addition to general topics of healthy nutrition (“What does a healthy diet look like? And why is a healthy diet important?”), this educational module focuses on the psychological aspects of eating (conscious and unconscious processes: Why do we eat, when and how? And how can we improve our eating behaviour?). This content is provided through lectures and exercises in individual and group work. It will have the same temporal scope (2 × 50 min) as the intervention module and will also take place in small groups and preferably in the same week of the pulmonary rehabilitation program.

At the beginning of the education module, the participants in the intervention group will receive a brochure with the education contents as well as supplementary information. Participants in the control group will receive this brochure after the 3-month follow-up. This way, the relevant education content is also made available to the control group. All participants also fill out a short and anonymous evaluation form, to be able to control the quality of the education modules from the participants’ point of view.

### Outcomes and measures

The primary and secondary outcomes are described in more detail below. Table 2 shows an overview of all measures and their survey times (which, when and how).

#### Primary outcomes

Data on asthma self-management at work as well as behavioural intention regarding asthma self-management at work (and workplace-related self-efficacy, see below) are collected in the same questionnaire.

##### Asthma self-management at work

Self-management is measured by a questionnaire devised [4] and previously used by our group [6]. It consists of ten items on trigger avoidance (1 item), acute symptom management (7 items, e.g., reliever medication use, breathing techniques, or taking breaks), and communication (2 items, referred to self-disclosure). For example, the following item relates to acute symptom management: “When my asthma at work is triggered by a certain situation, I leave that situation.” (for the full questionnaire, see Heinrichs et al., 2019 [6]). The response categories include the following: “Yes, I do this”, “No, but I would like to”, and “No, I do not need this”.

##### Behavioural intention

Behavioural intention is intended to measure the willingness to implement (for the first time or continued) self-management strategies in the workplace. For each self-management item, we ask the following in this context: “I plan to do this in the future (for the first time or continue).” Response categories include a 5-point Likert scale ranging from − 2 (“does not apply at all”) to + 2 (“fully applies”).

#### Secondary outcomes

##### Self-efficacy

Job-related self-efficacy is conceptualized in this study as referring to the belief that one feels capable of implementing self-management strategies at work as desired by oneself. For each item of the three subscales (trigger avoidance, acute symptom management, communication), we developed an associated item on self-efficacy: “I trust myself to do this in the future (for the first time or further).” The response categories correspond to those for behavioural intention (see above).

##### Self-management-related knowledge

Newly acquired knowledge of asthma self-management at work will be assessed by 20 items (potential score: 0–20). The items relate to the content of the intervention module and can be divided into four subscales: (1) asthma self-management at work in general, (2) rights as an employee, (3) job crafting, and (4) successful communication (with supervisors and colleagues). Each item is to be answered with “true” or “false”.

##### Number of asthma-related sick days

To examine the number of days of incapacity, we will ask the following question: “How many working days within the last three months (T3: since the end of rehabilitation) were you not able to work due to your asthma and… a)…called in sick (without a sick note)? b)…got a sick note from your physician?”

##### Number of asthma exacerbations

To examine the number of exacerbations, we will ask the following questions: “In the last three months (T3: since the end of rehabilitation), have you had at least one asthma attack (significant shortness of breath and use of reliever medication)?” and “In the last three months (T3: since the end of rehabilitation), have you experienced at least one significant worsening of your asthma over at least a few days (‘acute worsening’, ‘exacerbation’)?”

##### Asthma control

Asthma control will be measured by the well-established “Asthma Control Test” (ACT), which is a reliable and valid tool to identify patients with poorly controlled asthma [32]. The ACT is a 5-item instrument assessing asthma symptoms, use of reliever medication, and the effect of asthma on daily functioning (e.g., “In the past 4 weeks, how much of the time did your asthma keep you from getting as much done at work, school or at home?”). The ACT’s potential score ranges from 5 (very poorly controlled) to 25 (completely controlled) with higher scores indicating better control. A score of 19 or less has been defined as a cut-off score suggesting poor control [32].

##### Asthma-related quality of life

The “Marks Asthma Quality of Life Questionnaire” (AQLQ-M) includes 20 items that can be divided into 4 domains and sub scores: (1) breathlessness, (2) mood disturbance, (3) social disruption, and (4) health concerns. Individual items are scored from 0 (“not at all”) to 4 (“very severely”). In total scoring the AQLQ-M, higher scores represent a greater impact of asthma on quality of life. Internal consistency has shown to be high (Cronbach’s alpha for the total score of 0.92) and test-retest reliability has been shown to be adequate [33].

##### Subjective employment prognosis

The “Brief Scale Measuring the Subjective Prognosis of Gainful Employment” (SPE-scale) consists of three items with the following contents: (1) the expectation of being able to work until reaching the statutory retirement age due to the current state of health, (2) the permanent (subjective) threat to earning capacity due to the current state of health, and (3) the current thought of applying for a pension. The internal consistency and predictive validity could be confirmed [34, 35].

**Table 2:** Measures and survey times

Outcome	Measured by/Instrument	T0– screening	T1– rehabilitation start	T2– rehabilitation end	T3– 3-month follow-up
*Screening question*: Diagnosis “asthma” made by a physician, before the start of the COVID 19 pandemic in march 2020^1,2^	Self-developed	X			
*Screening question*: Employed for at least six months in an employment relationship subject to social insurance contributions or civil servant^1^	Self-developed	X			
*Screening question*: Working at least 15 h a week^1^	Self-developed	X			
Demographic information^3,4^	Self-developed	X	X	X	X
Asthma trigger	Self-developed		X	X	X
Sick days	Self-developed		X		X
Exacerbations	Partly adopted from “EPRA”^5^ -study [31]		X	X^9^	X
Information on treatment	Self-developed		X		X
Asthma self-management	Partly adopted from “EPRA”^5^-study [31]		X	X	X
Need for medical-occupational rehabilitation (MBOR)	Würzburger Screening [36]	X	X	X	Partly
Subjective prognosis of gainful employment	Brief Scale Measuring the Subjective Prognosis of Gainful Employment [34]		X	X	X
Information on occupation	Self-developed		X		X
Work satisfaction	Self-developed		X	X	X
Workability	First item from Work Ability Index [37]		X	X	X
Working conditions (related to asthma)	Determinants of Work-related Asthma Self-management Questionnaire [6]		X	X	X
Changes in working conditions	Self-developed				X
Asthma self-management at work (primary outcome T3)	Self-developed [6]		X	X	X
Behavioural intention regarding asthma self-management at work (primary outcome T2)	Self-developed		X	X	X
Workplace-related self-efficacy	Self-developed		X	X	X
Self-management-related knowledge (on “Asthma and Work”)	Self-developed		X	X	X
Job decision latitude	Copenhagen Psychosocial Questionnaire [38]		X	X	X
Social support at work	Copenhagen Psychosocial Questionnaire [38]		X	X	X
Asthma control	Asthma Control Test [32]	X	X	X	X
Quality of Life	Marks Asthma Quality of Life Questionnaire [33]		X	X	X
	Visual analog scale from European Quality of Life 5 Dimensions Questionnaire [39]		X	X	X
COVID-19	Self-developed/partly adopted from COVIDOM-study^6^ [40]		X	X^9^	X
Further health aspects^7^	Partly adopted from “EPRA”^5^-study [31]		X	X	X
Depressiveness	Patient Health Questionnaire-2 [41]		X	X	X
Anxiety	Generalized Anxiety Disorder Questionnaire-2 [41]		X	X	X
Body Mass Index^8^	Weight, height		X		
Lung function^8^	Forced expiratory volume in 1 s (FEV1), FEV1/vital capacity, total specific airway resistance, vital capacity, residual volume		X	X	
Exercise capacity^8^	6-minute walk test [42]		X	X	
Allergy^8^	Total immunoglobulin E (Ig E), specific Ig E-Screening (ImmunoCAP®)		X		
Asthmatic inflammation^8^	Fractional exhaled nitric oxide (FeNO), Eosinophils/µl		X		
Medication^9^	Asthma medication and changes		X	X	

^1^Inclusion criterion

^2^To ensure that asthma, not long/post COVID is the leading diagnosis

^3^Items vary slightly at different measurement time points

^4^Covariates of univariate analyses of covariance

^5^Effectiveness of Pneumological Rehabilitation in Bronchial Asthma

^6^Population-representative study of the sequelaes of COVID-19

^7^Includes physical activity, smoking behaviour, and doing relaxation procedures

^8^Assessed by a study nurse (with the help of a physician questionnaire)

^9^Assessed by a physician (with the help of a physician questionnaire)

### Data analysis plan

The primary statistical analysis will be based on the intention-to-treat (ITT) sample, that is, all participants as randomized. Since the ITT includes all participants for whom treatment was intended, data from individuals who, for example, did not ultimately receive the intervention or did not receive it completely (e.g., due to premature drop-out) are also included in the evaluation. Thus, the evaluation strategy based on the ITT principle is assumed to resemble outcomes that may be achieved in everyday life. Therefore, a secondary analysis based on the per-protocol collective (PP) will be performed. Here, all participants are excluded for whom the study treatment deviated from the study protocol (e.g., no participation in session 1 and/or 2). Since the PP only includes those participants who completed the study in compliance with the study protocol, the results may be biased in favour of the intervention. If the evaluation of the primary outcomes (see above) according to the ITT and PP principles provides similar results, confidence in the reliability of findings increases. If this is not the case, then possible reasons for the discrepancy between the two approaches will be discussed [43, 44].

The following describes the analyses in detail: Descriptive statistics, including means for continuous variables and proportions for categorical variables, will be used to summarize participant characteristics. We will determine the potential changes in primary and secondary outcomes (T1 to T2, T1 to T3) by pre-post comparisons within groups and test them for statistical significance using paired t-tests. Differences between groups (at T2 and T3) will be evaluated using univariate analyses of covariance (ANCOVAs). That is, membership in the intervention or control group will be used as the independent variable in each case, and the follow-up score of the primary outcome measures (behavioural intention, asthma self-management at work) and secondary outcome measures (self-efficacy, self-management-related knowledge, number of sick days, number of exacerbations, asthma control, asthma-related quality of life, and subjective employment prognosis) will be used as the dependent variable. Initially, only the corresponding baseline value of the primary outcome variable is included as a covariate to remove conditional bias [45, 46]. In a next step, the rehabilitation clinic [47], gender, age [48], and education [48] are additionally included as covariates. If the correlation between baseline and follow-up values is high (> 0.5), change scores are used as independent variables instead of follow-up values, as these then provide a more precise estimate [49]. For all models, the statistical model assumptions (linearity, homoscedasticity, normality of residuals) are tested. If the model assumptions are violated, appropriate analysis methods (e.g., robust regression, log transformation) are applied. In addition, to test whether comorbid COPD or long/post COVID disease affects the outcomes, sensitivity analyses will be performed excluding participants with COPD and/or long/post COVID symptoms. We will also conduct the analyses again without those who participated during the pre-test^2^, as at this point there may not yet be a routine regarding the training procedures and minor adjustments may still be made. Gender-stratified analyses are also conducted. Likewise, those with uncontrolled (ACT < 20 [32]) versus controlled asthma (ACT ≥ 20 [32]) and those with versus without a need for medical-occupational rehabilitation^3^ should additionally be analysed separately. Our primary analysis will be based on complete case analysis. In case the proportion of missing data exceeds 5% on a given variable, we will carry out sensitivity analysis employing multiple imputations.

Furthermore, the extent to which effects that may be observed are clinically relevant will be considered. In this context, the concept of “minimal clinically important difference” (MCID) is relevant. The MCID refers to the smallest change (i.e., improvement) that might be relevant to patients [50, 51]. Among other methods, the MCID can be estimated based on distribution. These include calculating MCID based on observed change, e.g., 1/2 standard deviation of change score [52]. This method has been recommended, particularly for smaller effects [52], and will therefore be used in the present study to assess whether potentially observed effects are clinically relevant.

## Discussion

Observational data suggested that psychosocial working conditions are associated with asthma self-management at work among employed individuals who are undergoing inpatient rehabilitation for asthma [4, 5]. Key working conditions included social support and job decision latitude, i.e., the extent to which work tasks and times can be determined at one’s own discretion [4, 5]. To our knowledge, experimental data from pulmonary rehabilitation on this issue are lacking. The aim of this study is therefore to develop and implement a novel patient education module that seeks to empower individuals with asthma to modify their working conditions in order to maximize their opportunities for appropriate asthma self-management at work.

Based on the assumption that the expected effects are observed, the patient education module could increase, amongst others, work ability, and social participation, improve return to work or staying at work, and reduce the number of sick days by improving asthma self-management as well as asthma control and asthma-related quality of life. Improving asthma self-management at work, but also asthma control and asthma-related quality of life [6, 7], could reduce costs associated with, e.g., asthma-related disability and early retirement. Ultimately, a patient education module should be available that is not only applicable to rehabilitation patients with asthma, but could also be evaluated in outpatient settings (e.g., disease management programs) or adapted to other chronic conditions.

### Electronic supplementary material

Below is the link to the electronic supplementary material.

Supplementary Material 1: SPIRIT reporting guidlines

## Data Availability

No datasets were generated or analysed during the current study.

## Footnotes

1 To ensure that asthma, not long/post COVID is the leading diagnosis.

2 Month 1 of the intervention period will serve as a pre-test: There will be two rounds of feedback from the teaching psychologists to the cross-site team (approximately after 2 and 4 weeks). If necessary, adjustments to the education modules will take place afterwards.

3 Medical-occupational rehabilitation focuses on the challenges of everyday working life. The need has been assessed by self-report (Würzburger Screening [36]).

## Abbreviations

ACT: Asthma Control Test
ANCOVA: Analyses of covariance
AQLQ–M: Marks Asthma Quality of Life Questionnaire
COPD: Chronic Obstructive Pulmonary Disease
FEV1: Forced expiratory volume in 1 s
FeNO: Fractional exhaled nitric oxide
IASUM: Institute for Occupational, Social and Environmental Medicine
ICD: International Statistical Classification of Diseases and Related Health Problems
ITT: Intention-to-treat
MBOR: Medical-occupational rehabilitation
MCID: Minimal clinically important difference
PP: Per protocol
RCT: Randomized controlled trial
SPE–scale: Brief Scale Measuring the Subjective Prognosis of Gainful Employment
sR_tot_: Total specific airway resistance
Ig E: Total immunoglobulin E
TPB: Theory of Planned Behaviour
VC: Vital capacity
